# Anharmonic Molecular Motion Drives Resonance Energy Transfer in *peri-*Arylene Dyads

**DOI:** 10.3389/fchem.2020.579166

**Published:** 2020-11-23

**Authors:** Vladislav Sláma, Václav Perlík, Heinz Langhals, Andreas Walter, Tomáš Mančal, Jürgen Hauer, František Šanda

**Affiliations:** ^1^Institute of Physics, Faculty of Mathematics and Physics, Charles University, Prague, Czechia; ^2^Department of Chemistry, Ludwig-Maximilians-Universität München, Munich, Germany; ^3^Professur für Dynamische Spektroskopien, Fakultät für Chemie, Technische Universität München, Munich, Germany

**Keywords:** perylene dyads, vibronic transport, MD/QC, Förster transport, ultrafast energy transfer

## Abstract

Spectral and dynamical properties of molecular donor-acceptor systems strongly depend on the steric arrangement of the constituents with exciton coupling *J* as a key control parameter. In the present work we study two *peri-*arylene based dyads with orthogonal and parallel transition dipoles for donor and acceptor moieties, respectively. We show that the anharmonic multi-well character of the orthogonal dyad's intramolecular potential explains findings from both stationary and time-resolved absorption experiments. While for a parallel dyad, standard quantum chemical estimates of *J* at 0 K are in good agreement with experimental observations, *J* becomes vanishingly small for the orthogonal dyad, in contrast to its ultrafast experimental transfer times. This discrepancy is not resolved even by accounting for harmonic fluctuations along normal coordinates. We resolve this problem by supplementing quantum chemical approaches with dynamical sampling of fluctuating geometries. In contrast to the moderate Gaussian fluctuations of *J* for the parallel dyad, fluctuations for the orthogonal dyad are found to follow non-Gaussian statistics leading to significantly higher effective *J* in good agreement with experimental observations. In effort to apply a unified framework for treating the dynamics of optical coherence and excitonic populations of both dyads, we employ a vibronic approach treating electronic and selected vibrational degrees on an equal footing. This vibronic model is used to model absorption and fluorescence spectra as well as donor-acceptor transport dynamics and covers the more traditional categories of Förster and Redfield transport as limiting cases.

## 1. Introduction

*Peri*-arylenes and their heterodimers are suitable model systems to study excitation energy transfer due to their chemical versatility and convenient spectroscopic properties, such as high fluorescence quantum yield and photostability (Langhals, 2005, 2013). They were applied as laser dyes (Löhmannsröben and Langhals, 1989; Qian et al., 2003), fluorescent light collectors (Garvin, 1960; Seybold and Wagenblast, 1989; Langhals et al., 1998; Kalinin et al., 2001; Langhals, 2019), fluorescent probes (Bo et al., 2013), or fluorophores for single-molecule spectroscopy (Mais et al., 1997; Lang et al., 2005). Their significant charge transport abilities (Huang et al., 2011) can be extended over larger aggregates, which makes them suitable building blocks for organic photovoltaics (Hofmann et al., 2010; Holcombe et al., 2011). The family of *peri-*arylenes (also called rylenes), e.g., perylene, terrylene, and their chemical derivatives, exhibits similar spectroscopic properties (Herrmann and Müllen, 2006) such as fluorescence lifetimes around 5 ns with quantum yield near unity. Their absorption and fluorescence spectra are dominated by a transition between the highest occupied and the lowest unoccupied molecular orbital in the visible spectral region with characteristically strong vibronic progression peaks arising from a ring stretching mode around 1, 400 cm^−1^ (Ambrosino and Califano, 1965).

*Peri*-arylenes can be chemically linked to form dyads, the spectral features and excitation energy transfer dynamics of which are fine-tuned by their relative geometric arrangement and side group substitutions (Langhals and Gold, 1996, 1997; Langhals and Saulich, 2002; Osswald and Würthner, 2007; Fron et al., 2008). These structural parameters are readily modified by well-established synthetic routes (Langhals, 2013), leading to a large variation of intermolecular coupling in different *peri-*arylene dyads. Accordingly, optical response and excitation transfer between donor and acceptor moieties may occur under weak intermolecular coupling (Würthner et al., 2001) described by Förster theory (Förster, 1948) or in a scenario where the intermolecular coupling dominates over electron-vibrational modulations described by Redfield theory (Redfield, 1957). Various approaches have been developed to interpolate between these limits (Šanda and Mukamel, 2006, 2008; Tanimura, 2006; Zimanyi and Silbey, 2012; Fujihashi and Kimura, 2013). Regardless wide range of realizable coupling strengths in dyadic systems, a common feature in all *peri-*arylenes is the aforementioned pronounced vibronic progression in their spectra, representing strong modulation of electronic transition by an underdamped ring stretching mode. Such a scenario implies to treat this vibration on an equal footing with electronic degrees of freedom (Polyutov et al., 2012; Butkus et al., 2014; Perlík et al., 2014; Hestand and Spano, 2018). The effects of the remaining vibrations are already moderate and their perturbative treatment can be justified for a broad family of *peri-*arylene dyadic systems.

**Figure 1:**
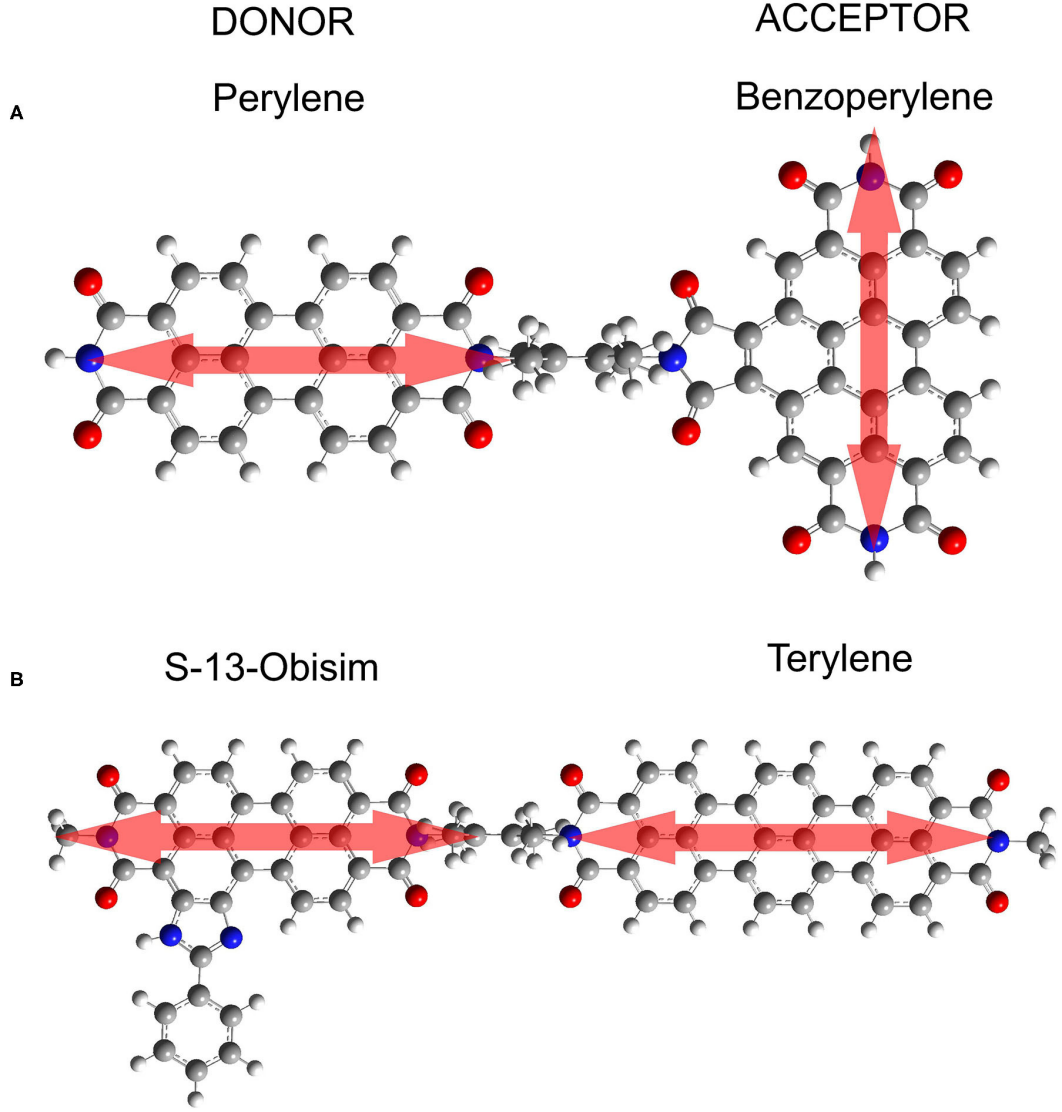
Simulated donor-acceptor systems. **(A)** Perylene-benzoperylene bisimide dyad in orthogonal arrangement. The distance between centers of the transition charge centroids is 16 Angstrom. **(B)** S-13-obisim-terrylene dyad in parallel arrangement.

In the present work, we use this vibronic approach (Perlík and Šanda, 2017) to unify the treatment of two *peri-*arylene dyads with parallel and orthogonal transition dipole moments of donor and acceptor, as depicted in Figure 1. The parallel arrangement leads to coupling strengths beyond the Förster limit, while the geometry of the orthogonal dyad suggests very weak interchromophoric coupling. Within the vibronic framework, we reproduce the absorption and fluorescence spectra and demonstrate that the employed vibronic approach tracks excitation energy transfer through the entire energy ladder of vibronic states for both marginal and strong couplings.

The speed of donor-to-acceptor transport is primarily determined by donor-acceptor coupling constant *J*. The ultrafast transfer times reported in literature are thus one primary source of *J* estimates. Alternatively, the value of *J* can be derived from a microscopic model using quantum chemical methods. The orthogonal dyad was reported (Langhals et al., 2010; Langhals and Walter, 2020) to represent a challenging case in this context: Despite the apparent lack of coupling in the optimal ground state geometry, transient absorption experiments show transfer time in the sub-10 ps range. This apparent discrepancy was attributed to thermally induced deviations from a strictly orthogonal molecular geometry. While this hypothesis was supported by temperature- and solvent-dependent experiments (Langhals et al., 2010; Nalbach et al., 2012), a microscopic understanding of the observed transfer times and coupling strengths is still missing; normal mode fluctuation analysis of the intra-dyad coupling underestimated the coupling strength by an order of magnitude. We resolve this problem by supplementing a microscopic density functional theory (DFT) parametrization with molecular dynamics (MD) sampling of molecular geometries. We demonstrate that the intramolecular potential has a multi-well character, and that the typical geometry at room temperature deviates significantly from the minimal energy geometry. This anharmonic character is not captured by a standard linearization of forces around the global potential minimum, which explains why harmonic approaches consistently underestimate *J* in the orthogonal dyad.

Hence, we present how a vibronic approach describes absorption and fluorescence spectra of the two *peri-*arylene dyads in vastly different coupling regimes and how the model can be parameterized by a microscopic MD/DFT approach, which in the case of the orthogonal dyad uncovers the anharmonic character of the potential landscape determining *J*.

The paper is organized as follows: In section 2, we describe the methods used to obtain the quantum chemical estimates of couplings at finite temperatures and beyond the dipole-dipole approximation (section 2.1) and introduce the vibronic model of optical dynamics (section 2.2). We also give brief accounts of sample preparation and spectral measurements. In section 3, we fit the absorption and fluorescence spectra of both dyads using a vibronic model and determine *J* of the parallel dyad (section 3.1). The population dynamics for both dyads are discussed and an effective coupling for the orthogonal dyad is determined in section 3.2. We then proceed (section 3.3) to test quantum chemical calculations of *J* and demonstrate that the entire configuration space of the orthogonal dyad must be sampled to obtain estimates for *J* in agreement with experimental observations. In section 4, we conclude.

## 2. Methods

### 2.1. Estimates of Intermolecular Coupling

Dynamics of the optical coherence of the investigated dyads are induced by interaction (${\hat{V}}_{AD}$) between the electronic structures of donor (${\hat{H}}_{el}^{D}$) and acceptor (${\hat{H}}_{el}^{A}$) and are further modulated by dynamics of nuclei (Ĥ_*nc*_)

(1) $$\begin{array}{l} \hat{H}={\hat{H}}_{el}^{A}+{\hat{H}}_{el}^{D}+{\hat{H}}_{nc}+{\hat{V}}_{AD}. \end{array}$$

In the Born-Oppenheimer approximation electronic and nuclear motions are separated (Atkins and Friedman, 2011). In the following we refer to the local instantaneous electronic eigenstates (*k* = 0, 1, … ) satisfying

(2) $$\begin{array}{l} {\hat{H}}_{el,R}^{A}{\text{Ψ}}_{k,R}^{A}\left(r_{A}\right)=E_{k,R}^{A}{\text{Ψ}}_{k,R}^{A}\left(r_{A}\right) \end{array}$$

where ***r***_*A*_ = (*r*_1_, *r*_2_, …) is a collection of electronic coordinates on the acceptor, and the parameter ***R*** stands for molecular geometry specified by a collection of nuclear coordinates. The complete information of the many-body wavefunction is excessively abundant, and for most purposes is reduced to the one-particle transition densities (McWeeny, 1960) between the electronic states *k* and *k*′ defined as

(3) $$\begin{array}{l} \begin{array}{c} \rho_{kk^{\prime },R}^{A}\left(r\right)=\int {\text{Ψ}}_{k,R}^{A}\left(r_{1},r_{2},\ldots \right){\text{Ψ}}_{k^{\prime },R}^{*A}\left(r_{1},r_{2},\ldots \right)\\ \;\times \underset{j\in \left\{A\right\}}{\sum }\delta \left(r-r_{j}\right)\underset{i\in \left\{A\right\}}{\prod }dr_{i}. \end{array} \end{array}$$

The donor variables *E*^*D*^, ***r***_*D*_, Ψ^*D*^, ρ^*D*^ are defined analogously.

Interchromophore coupling accounts for electrostatic interaction of electronic coordinates *r*_*i*_ of, e.g., the acceptor (*i* ∈ {*A*}) with those of donor (*j* ∈ {*D*}) and for their interaction with nuclear coordinates *R*_*J*_ (of proton number *Z*_*J*_) outside the acceptor (including a linker)

(4) $$\begin{array}{l} {\hat{V}}_{AD}=\underset{\begin{array}{c} i\in \left\{A\right\}\\ j\in \left\{D\right\} \end{array}}{\sum }\frac{1}{\left|r_{i}-r_{j}\right|}-\underset{\begin{array}{c} i\in \left\{A\right\}\\ J\notin \left\{A\right\} \end{array}}{\sum }\frac{Z_{J}}{\left|r_{i}-R_{J}\right|}-\underset{\begin{array}{c} I\notin \left\{D\right\}\\ j\in \left\{D\right\} \end{array}}{\sum }\frac{Z_{I}}{\left|R_{I}-r_{j}\right|}. \end{array}$$

Intermolecular exciton transfer occurs predominantly between the two lowest excited states $|\Psi_{1,R}^{A}\rangle |\Psi_{0,R}^{D}\rangle$ and $|\Psi_{0,R}^{A}\rangle |\Psi_{1,R}^{D}\rangle$. Resonance coupling between them

(5) $$\begin{array}{l} J_{R}\equiv \langle {\text{Ψ}}_{1,R}^{A}|\langle {\text{Ψ}}_{0,R}^{D}|{\hat{V}}_{AD}|{\text{Ψ}}_{0,R}^{A}\rangle |{\text{Ψ}}_{1,R}^{D}\rangle \end{array}$$

can be recast in terms of transition densities as

(6) $$\begin{array}{l} J_{R}=\int \frac{\rho_{01,R}^{A}\left(r_{a}\right)\rho_{01,R}^{D}\left(r_{d}\right)}{\left|r_{a}-r_{d}\right|}dr_{a}dr_{d}. \end{array}$$

The direct discretization of Equation (6) known as the transition density cube (TDC) method (Krueger et al., 1998) shall be used at short intermolecular distances. When the molecules are far apart (with respect to molecular size), Equation (6) is well-approximated by classical interaction between point dipoles ${\vec{\mu }}_{R}^{A\left(D\right)}\equiv \int \vec{r}\rho_{01,R}^{A\left(D\right)}\left(r\right)dr$.

At finite temperatures 1/*k*_*B*_β, electronic structures and couplings depend on the molecular geometry sampled along the Boltzmann distribution with expectation values 〈… 〉 obeying

(7) $$\begin{array}{l} \langle f\rangle \equiv \frac{\int f\left(R\right)e^{-\beta E\left(R\right)}\text{d}R}{\int e^{-\beta E\left(R\right)}\text{d}R}. \end{array}$$

Here, we evaluate the ground state energies

(8) $$\begin{array}{l} E\left(R\right)\equiv \langle {\text{Ψ}}_{0,R}^{A}|\langle {\text{Ψ}}_{0,R}^{D}|\hat{H}|{\text{Ψ}}_{0,R}^{A}\rangle |{\text{Ψ}}_{0,R}^{D}\rangle \end{array}$$

using density functional theory (DFT) which draws upon recasting Equation (8) in the form of Kohn-Sham functional (Parr and Yang, 1989). In particular, we evaluated *E*(*R*) using GAUSSIAN 09 quantum chemistry package (Frisch et al., 2009) at DFT level with B3LYP functional and 6-311(d,p) basis set.

For calculation of the transition densities $\rho_{01}^{D\left(A\right)}$ of donor (acceptor) the linker molecule was replaced by a methyl group, and TD-DFT was employed using long range corrected CAM-B3LYP functional, which is known to provide correct (experimental) excitation energies for aromatic hydrocarbon derivatives (de França et al., 2018).

The coupling estimates are usually feasible for a zero temperature geometry ***R***_0_, characterized by the minimal ground state energy *E*(***R***_0_) ≤ *E*(***R***). For the orthogonal dyad, however, *J*_***R***_0__ ≈ 0, and the thermal fluctuations might dominate the effective value of transport. We thus explore distributions of coupling 〈δ(*J* − *J*_***R***_)〉. To avoid costly complete exploration of high dimensional ***R*** configuration space, we compare sampling by normal mode analysis (NMA) and by molecular dynamics (MD).

In the NMA, energy is expanded to second order $E\left(R\right)=E\left(R_{0}\right)+\underset{I,J}{\sum }\varepsilon_{IJ}\left(R_{I}-R_{0,I}\right)\left(R_{J}-R_{0,J}\right)$ around the minimum. The Hessian $\varepsilon_{IJ}\equiv \frac{\partial E}{\partial R_{I}\partial R_{J}}$ is diagonalized to obtain the normal mode coordinates. Geometry sampling is restricted along a single normal coordinate, otherwise weighted according to Equation (7), and the couplings on the samples are evaluated using TDC (Equation 6).

However, the NMA ignores the complexity of potential surfaces. An alternative approach is thus to employ MD to sample molecular geometries, avoiding also the harmonic approximation to *E*(***R***) inherent to NMA. Using the AMBER package (Case et al., 2014) with GAFF force field (Wang et al., 2004) and RESP charges (Bayly et al., 1993) calculated by Gaussian09 software (Frisch et al., 2009) the conformation space sampling was performed for an NpT ensemble of a single *peri-*arylene dyad with 2,697 toluene solvent molecules at atmospheric pressure and room temperature of 300 K and a 0.2 fs time-step was used for numerical integration of equations of motion. After an initial 10 ns for equilibration, the dyad geometries were sampled at 40 fs frequency and used for a QC calculation of resonance coupling.

In transport simulations, such as below, costly sampling of coupling distributions is usually avoided by representing coupling as a single effective value. In most situations, thermal fluctuations are minor |〈*J*^2^〉 − 〈*J*〉^2^| ≪ 〈*J*〉^2^ and the effective coupling should be compared with the mean value, which often corresponds to that of **R**_0_ geometry *J* ≡ 〈*J*〉 ≈ *J*_**R**_0__. At the other extreme, major fluctuations $\langle J\rangle <\sqrt{\langle J^{2}\rangle }$, which are applicable to the orthogonal dyad, where the geometric symmetries require 〈*J*〉 = 0 (and symmetric coupling distributions), the coupling distribution should be effectively represented by $J\equiv \sqrt{\langle J^{2}\rangle }$, which scales with ∝ *J*^2^, as expected for Förster transfer.

### 2.2. Vibronic Model for *peri-*arylene Dyads

What remains is to connect the microscopic parameterizations of the previous section to the vibronic dynamics of Perlík and Šanda (2017). To this end, we formally define excitonic states and energies by fixing $H_{el}^{A\left(D\right)}$ at typical geometry ***R***_0_. To model absorption and fluorescence spectra as well as population dynamics, the relevant states are: the ground state $|g\rangle \equiv |\Psi_{0,R_{0}}^{A}\rangle |\Psi_{0,R_{0}}^{D}\rangle$ and two singly excited states $|e_{A}\rangle \equiv |\Psi_{1,R_{0}}^{A}\rangle |\Psi_{0,R_{0}}^{D}\rangle$ and $|e_{D}\rangle \equiv |\Psi_{0,R_{0}}^{A}\rangle |\Psi_{1,R_{0}}^{D}\rangle$. To complete the Frenkel exciton Hamiltonian the effective intermolecular coupling *J* between |*e*_*A*_〉 and |*e*_*D*_〉 is added (other elements of ${\hat{V}}_{AD}$ are neglected)

(9) $${\hat{H}}_{F}=\epsilon^{A}|e_{A}\rangle \langle e_{A}|+\epsilon^{D}|e_{D}\rangle \langle e_{D}|+J(|e_{A}\rangle \langle e_{D}|+|e_{D}\rangle \langle e_{A}|)$$

where $\epsilon^{A}\equiv E_{1,R_{0}}^{A}-E_{0,R_{0}}^{A}$ and $\epsilon^{D}\equiv E_{1,R_{0}}^{D}-E_{0,R_{0}}^{D}$. We next introduce vibronic dynamics by separating from Ĥ_*nc*_ the underdamped ring stretching vibrations *q*_*A*_ of *peri-*arylenes around 1,400 cm^−1^

(10) $${\hat{H}}_{vib}^{A}=\frac{{\hat{p}}_{A}^{2}}{2m}+\sum_{\text{j}=0}^{1}V(q_{A}-d_{\text{j}}^{A})|\Psi_{\text{j},R_{0}}^{A}\rangle \langle \Psi_{\text{j},R_{0}}^{A}|,$$

where $d_{\text{j}}^{A}$ are displacements of electronic surface *V*(*q*) (vibrational states shall be labeled ν_*A*_ = 0, 1, … .) and introduce them into the system Hamiltonian Ĥ_*s*_

(11) $$\begin{array}{l} {\hat{H}}_{s}={\hat{H}}_{F}+{\hat{H}}_{vib}^{A}⊗{𝟙}^{D}+{𝟙}^{A}⊗{\hat{H}}_{vib}^{D}. \end{array}$$

Diagonalization of vibronic Hamiltonian Equation (11) explains the peak position and magnitudes in the absorption and fluorescence spectra.

Other low-frequency vibrations and solvent degrees of freedom affect line-widths and shapes. They are treated as bath fluctuations and their effect is represented by the magnitude λ_*V*_ (λ_*W*_) and inverse timescale Λ_*V*_ (Λ_*W*_) of linear (quadratic) stretch vibration-to-bath coupling (Caldeira and Leggett, 1983). Similar parameters λ_*U*_ and Λ_*U*_ are introduced for the magnitude and rate of electronic dephasing. Their microscopic definitions are summarized in Supplementary Material, section 1. These parameters are estimated by fitting the absorption spectrum, their microscopic calculations are principally possible but a challenging task (Olbrich and Kleinekathöfer, 2010; Olbrich et al., 2011; Renger et al., 2018).

The optical probes are described by the interaction Hamiltonian

(12) $${\hat{H}}_{i}=\mu_{A}(t)|e_{A}\rangle \langle g|+\mu_{D}(t)|e_{D}\rangle \langle g|+h.c.,$$

where $\mu_{A\left(D\right)}\left(t\right)\equiv {\vec{\mu }}_{A\left(D\right)}\cdot \vec{E}\left(t\right)$ is the projection of the laser field $\vec{E}\left(t\right)$ on the transition dipole ${\vec{\mu }}_{A}\left({\vec{\mu }}_{D}\right)$ between the *g* and *e* states of the acceptor (donor) at **R**_0_ geometry, respectively. The transition dipole dependencies on vibrational coordinates *q*_*A*_, *q*_*D*_ are usually neglected in the Condon approximation.

Details of the simulations, i.e., the quantum master equation to describe vibronic population transfer and second cumulants for line-shapes, were published previously (Perlík and Šanda, 2017). We also adopted correction factors Ω, and Ω^3^ to connect absorption and fluorescence spectra, respectively, with the response functions along (Angulo et al., 2006).

### 2.3. Sample Preparation

The preparation of the orthogonal dyad has been published previously (Langhals et al., 2008).

The parallel dyad was synthesized in two steps. The linker was first substituted at the terrylene molecule: Terrylene anhydride carboximide was solubilized by means of the long-chain secondary alkyl substituent 1-nonyldecyl at the nitrogen atom and condensed with an excess of 2,3,5,6-tetramethylbenzene-1,4-diamine (see Supplementary Material, section 3.1 for detail of synthesis). The free primary amino group of the thus obtained terrylenebiscarboximide was in the second step further condensed with an imidazoloperyleneanhydridecarboximde to obtain the dyad with a rigid, orthogonal spacer between the two chromophores for electronic decoupling, where the two peripheric *sec*-alkyl substituents render both a sufficiently high solubility and a low tendency for aggregation. Hence, any potential red-shift upon covalent linking of donor and acceptor moieties is due to electronic coupling and not due to aggregation effects (see Supplementary Material, section 3.2 for detail of synthesis).

### 2.4. Spectroscopic Methods

All absorption and fluorescence emission measurements on both investigated substances were performed in chloroform at an optical density (OD) of 0.35 at the respective absorption maxima at an optical path length of 1 cm. Absorption and fluorescence spectra were recorded at a resolution of 1 nm. Reabsorption of fluorescence light caused by the significant overlap of absorption and fluorescence spectra was corrected according to procedures described previously (Didraga et al., 2004; Lincoln et al., 2016).

## 3. Results

### 3.1. Absorption and Fluorescence Spectra

The absorption *S*(Ω) and fluorescence *F*(Ω) spectra of constituent *peri-*arylene subunits in the visible reveal a single electronic transition modulated by a strong vibronic progression peaks. They stem from electron couplings to a ring stretching mode around ω ≈ 1, 400 cm^−1^. The magnitudes of the progression peaks are significantly asymmetric between absorption and fluorescence *S*(ϵ + ω) ≠ *F*(ϵ − ω), which clearly rule out the standard harmonic model of vibration (Angulo et al., 2006). Thus, for the sake of simplicity we adopted the anharmonic oscillator model (Anda et al., 2016; Galestian Pour et al., 2017) $V\left(q\right)=\frac{1}{2}m\omega^{2}{\hat{q}}^{2}+\alpha {\hat{q}}^{3}$, while retaining the Born-Oppenheimer and the Condon approximations. The frequencies, displacement, and anharmonicities for this vibrational stretch mode are obtained by comparing positions and magnitudes of progression peaks of absorption and fluorescence following a previously established protocol (Galestian Pour et al., 2017). We also fit the spectral profiles and Stokes shift to obtain the bath parameters λ and Λ, which are summarized in Table 1. These constituent parameter fits are then fixed, and intermolecular coupling is varied to match the dyad absorption.

**Table 1:** Parametrization (in cm^−1^) of the vibronic model for the absorption and fluorescence spectra of the dyads constituents.

**Molecule dyad role**	**Perylene orthogonal donor**	**Benzoperylene orthogonal acceptor**	**S-13-obisim parallel donor**	**Terrylene parallel acceptor**
ω	1,470	1,420	1,370	1,380
*d*^2^*mω*/(2ℏ)	0.8	0.7	0.6	0.5
α	−35	−10	−13	6
*E*_1_−*E*_0_	21,650	19,100	17,000	15,450
Λ_*U*_	200	360	180	330
Λ_*V*_	200	270	40	30
Λ_*W*_	–	–	20	15
λ_*U*_	350	260	260	300
λ_*V*_	200	270	300	250
λ_*W*_	0	0	20	15
μ	0.8	1	0.8	0.9

**Figure 2:**
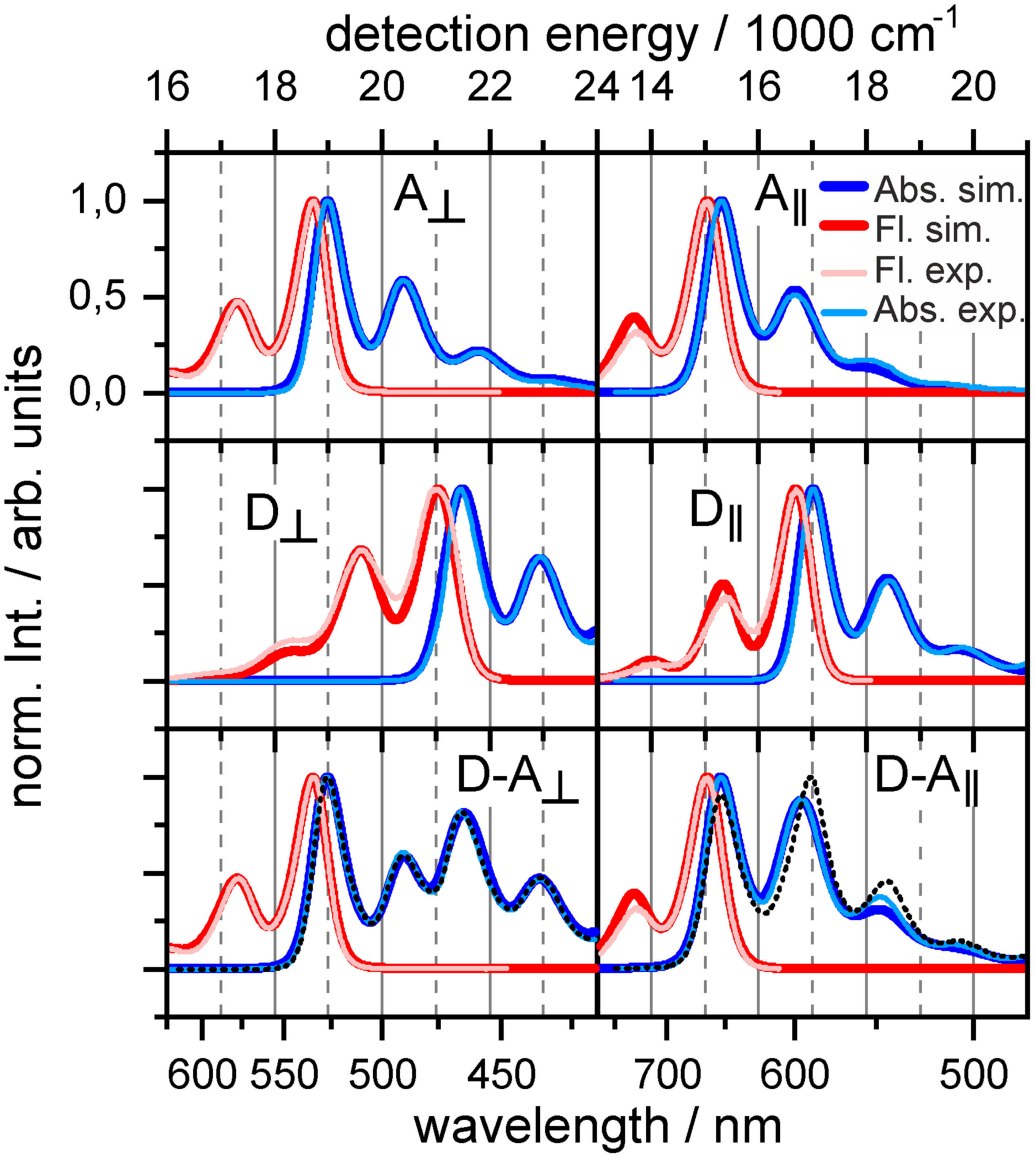
Experimental and simulated absorption (blue lines) and fluorescence (red lines) spectra for acceptor **(top)**, donor **(middle)**, and dyad **(bottom)**. The dashed black line combines absorption of donor and acceptor *S*_*A*_ + *S*_*D*_. Left: Orthogonal dyad. Right: Parallel dyad.

Comparison between the monomeric and dyadic absorption spectra indicates the parametric regime for each orientation. The absorption spectrum of the orthogonal dyad can be almost perfectly reconstructed by a simple addition of the donor and acceptor absorption lineshapes (see Figure 2, bottom left). The dyadic absorption spectrum thus does not provide means to quantify the magnitude of coupling *J*, but limits the parameters to the weak coupling regime consistent with Förster theory. In contrast, the difference between the absorption of the strongly coupled parallel dyad and its constituents (Figure 2, bottom right) sets *J* within the range of −150 to −250 cm^−1^ (Figure 2 shows best fit at *J* = −200*cm*^−1^), which suggests a departure from Förster theory.

### 3.2. Population Dynamics

In section 3.1, we have successfully simulated the linear optical responses of both dyads, however, the spectrum of the orthogonal dyad does not allow for a reliable determination of the value of *J*, other than that it must be in the weak coupling regime. Donor to acceptor transfer rates can be used instead to bring the comparison with experiment. Transient absorption measurement by Langhals et al. (Figure 2 in Langhals et al., 2010) have reported single lifetime of τ = 9.4 ps associated with transfer from donor to vibrationless acceptor. At early times around hundred femtosecond traces of vibrational relaxation are visible.

We have simulated the population evolution for the orthogonal dyad after the experimentally employed (Langhals et al., 2010) excitation at 23, 000 cm^−1^, which corresponds to the first donor excited vibrational state. We have used the full vibronic model parameterized from absorption/fluorescence of constituents as given in Table 1. The intermolecular coupling was varied to reproduce the experimentally determined transport dynamics best fitted at *J* = 16 cm^−1^.

**Figure 3:**
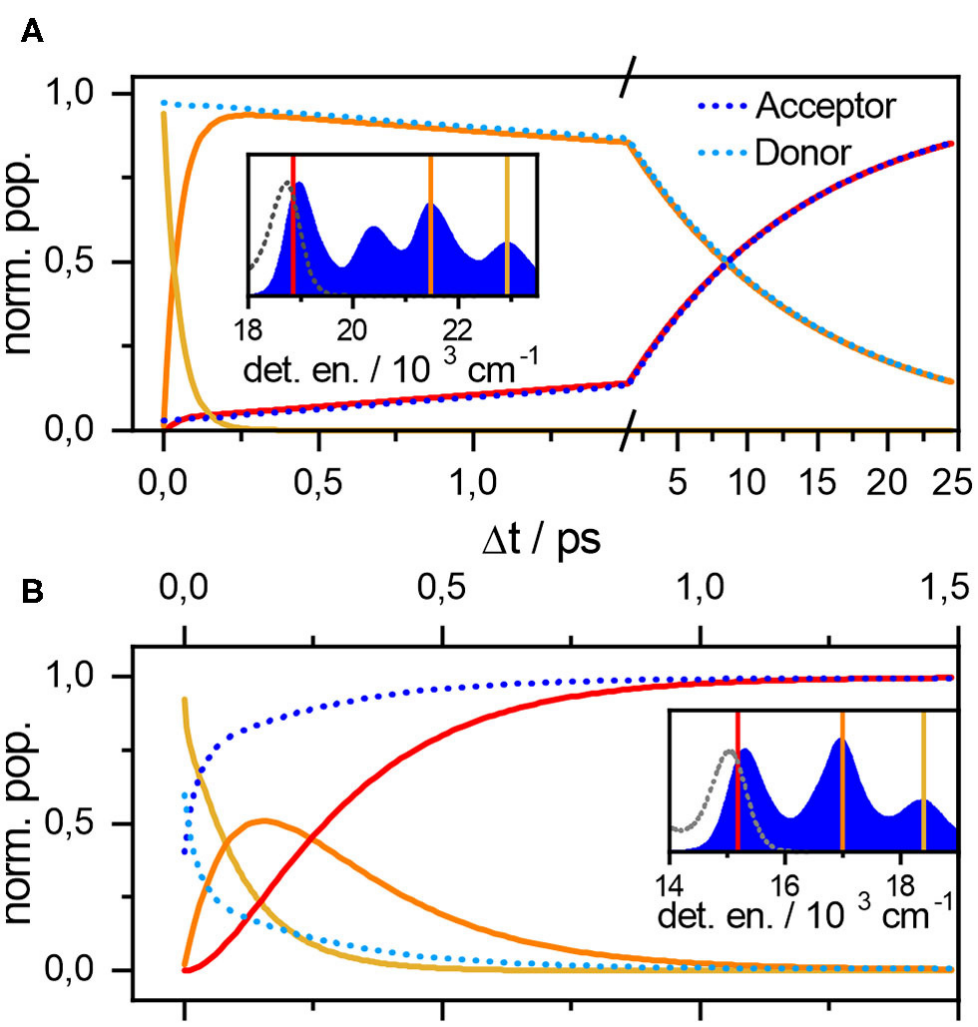
Population evolutions for the orthogonal **(A)** and parallel **(B)** dyad. Parameters are the same as for Figure 2. Population evolution of donor (light blue), acceptor (dark blue) states as well as populations of resonant levels around the color-coded energies. The inset shows these energies with respect to the absorption (full blue area) and emission spectrum (gray dashed lines).

Simulations (Figure 3, top) show early vibrational relaxation localized at the donor molecule around 100 fs, followed by slower exciton transport in picoseconds. The two timescales are thus well separated providing the standard picture of Förster type of transport in the usual experimental observation window >100 fs limited by narrowband excitation. To demonstrate the compatibility with the celebrated Förster formula

(13) $$\begin{array}{l} \frac{1}{\tau }=\frac{1}{\hbar^{2}c}|J{|}^{2}\int_{0}^{\infty }\frac{F_{D}\left(\Omega \right)S_{A}\left(\Omega \right)}{\Omega^{4}}d\Omega , \end{array}$$

we applied it using the aforementioned coupling constant *J* = 16 cm^−1^ to yield τ = 11ps, which compares well to the observed τ = 9.4ps. Alternatively, taken from the coupling point of view, Förster theory would require *J* = 17 cm^−1^ to correctly estimate the experimental lifetimes (Megerle et al., 2009; Langhals et al., 2010). This good agreement makes our vibronic treatment truly unified approach to *peri-*arylene aggregates as this essentially weak system-bath coupling perturbative scheme works well even for the weak intermolecular coupling (orthogonal) case.

The more complicated transport dynamics of the strongly coupled parallel dyad is depicted in lower panel of Figure 3. Transport has been simulated using the parameters for donor and acceptor moieties given in Table 1 and at a resonance coupling of *J* = −200 cm^−1^. The donor and acceptor principal transitions are shifted by approximately one vibrational quantum. The vibronic states are thus highly delocalized appearing at resonances ϵ^*D*^ + *nω* ≈ ϵ^*A*^ + (*n* + 1)ω. To understand the underlying transport dynamics through the vibronic energy ladder in detail, we simulated populations of the donor *P*^*D*^, the acceptor *P*^*A*^ (dotted lines), and the total populations of the resonance manifolds (solid lines in Figure 3).

The parallel dyad is assumed to be excited by a narrowband pulse at 18,250 cm^−1^ around the ϵ^*D*^ + ω ≈ ϵ^*A*^ + 2ω resonance, i.e. tuned to the first vibrational excited state of donor, ensuring the dynamics is comparable to that of the orthogonal dyad studied above. The initial population of acceptor *P*^*A*^(Δ*t* = 0) = 0.4 is only slightly lower than that of the donor *P*^*D*^(Δ*t* = 0) = 0.6 as a result of high delocalization. One vibrational quantum is lost within approximately 200 fs (yellow line in Figure 3B) to appear at lower ϵ^*D*^ ≈ ϵ^*A*^ + ω = 16, 500 cm^−1^ resonance (orange line). This transition corresponds to the early Δ*t* < 200 fs component of biexponential relaxation. The asymptotic component after 200 fs is seemingly connected with further relaxation from the resonance and final localization in the acceptor vibrational ground state |*e*_*A*_; ν_*A*_, ν_*D*_ = 0〉 at 15, 100cm^−1^ (red line). The relaxation is nevertheless completed within 1 ps as the exciton dynamics between these strongly coupled chromophores is significantly faster dynamics as compared to the orthogonal case. The two processes thus appear at quite similar timescales, providing the example of truly vibronic relaxation dynamics. The experimental confirmation of this behavior, and thus confirmation of *J* estimate, is a matter of ongoing research.

### 3.3. Distributions of the Intermolecular Coupling

Having established estimates for the intermolecular coupling *J* for both dyads based on fits to experimental data, we can now proceed to develop sound procedures for its quantum chemical calculations.

The DFT-optimized geometry ***R***_0_ of the dyad displayed at Figure 1A has orthogonal dipoles and vanishing coupling *J*_***R***_0__ = 0 (within numerical error) as expected from symmetry. We identified five lowest (i.e., thermally populated) normal modes which correspond to bending and torsion of the dyad (Langhals et al., 2010). Coupling distributions calculated at room temperature along each of these coordinates are approximately Gaussian with zero mean $\bar{J}\equiv \langle J\rangle \approx 0$. Typical values of coupling estimated as standard deviations $\sqrt{\langle J^{2}\rangle }$ are summarized in the Table 2. These values are an order of magnitude too small to explain the experimentally observed transfer times. In other words, no normal mode can be associated with the required coupling fluctuations.

**Table 2:** Five lowest normal modes and standard deviation of coupling distributions 〈*J*^2^〉 for orthogonal dyad.

**Normal mode**	**Frequency [cm^**−1**^]**	**$\sqrt{\langle J^{2}\rangle }$**
1	7.93	1.55
2	8.4	0.22
3	12.39	0.09
4	20.81	1.34
5	21.98	0.02

*In all cases 〈J〉 ≈ 0*.

**Figure 4:**
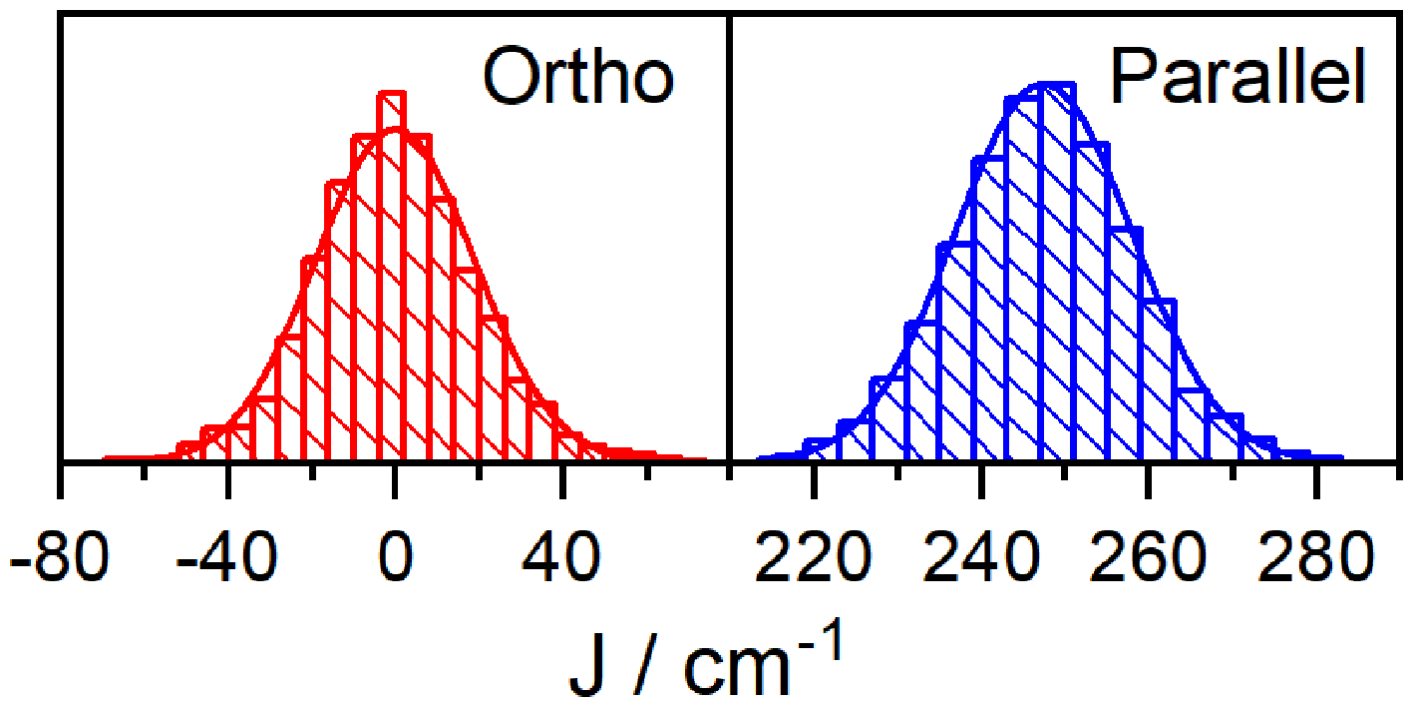
Histogram of coupling strengths for the orthogonal dyad **(left)**. Mean and standard deviations are 〈*J*〉 = 0cm^−1^, $\sqrt{\langle J^{2}\rangle -{\langle J\rangle }^{2}}=19$cm^−1^. **(Right)** Parallel dyad and 〈*J*〉 = −247cm^−1^, $\sqrt{\langle J^{2}\rangle -{\langle J\rangle }^{2}}=11$cm^−1^.

We now turn to an exploration of the complete *E*(***R***) landscapes. Regular samplings of the (high dimensional) configuration space are inefficient. We have thus used MD software package AMBER and sampled geometries with 40 fs time step after an initial 10 ns equilibration (when the geometry fluctuations become stable) at room temperature (300 K) in two runs (production times 80 ps, 40 ps) to check that trajectory is not biased. For each geometry, the coupling was estimated using the methods of section 2.1. The distributions for the orthogonal dyad, shown on the left panel of Figure 4, are—within statistical error—symmetric around *J* = 0. Nevertheless, the absolute values of the coupling are rather large, with median of |*J*| to be 12 cm^−1^ and a mean value 〈|*J*|〉 = 15 cm^−1^. In the context of Förster transport and its ∝ *J*^2^ scaling, the standard deviation $\sqrt{\langle J^{2}\rangle }=19{\text{cm}}^{-1}$ is the most meaningful measure for an effective coupling constant. Therefore, we observe an enhancement of the intermolecular coupling after MD analysis over the values obtained after NMA. This is surprising and calls for a detailed investigation. We noticed, that the distribution has thicker tails than Gaussian fit, this deviation has been quantified by estimating Pearson's kurtosis $\kappa_{J}=\frac{\langle {\left(J-\bar{J}\right)}^{4}\rangle }{{\langle {\left(J-\bar{J}\right)}^{2}\rangle }^{2}}=3.66$, large excess over κ = 3 of normal distribution. With this estimate, the distribution can be rejected to follow Gaussian statistics at standard 95% confidence level (test accounted for correlations appearing along MD trajectory, see Supplementary Material, section 2 for details).

Non-Gaussian statistics can represent anharmonic vibrational motions in complex multi-well potential landscapes. To underline the evidence of anharmonic effects, we studied longer MD trajectories until 800 ps (20,000 configurations with 40 fs time-step) at various temperatures, while avoiding costly QC calculations.

**Figure 5:**
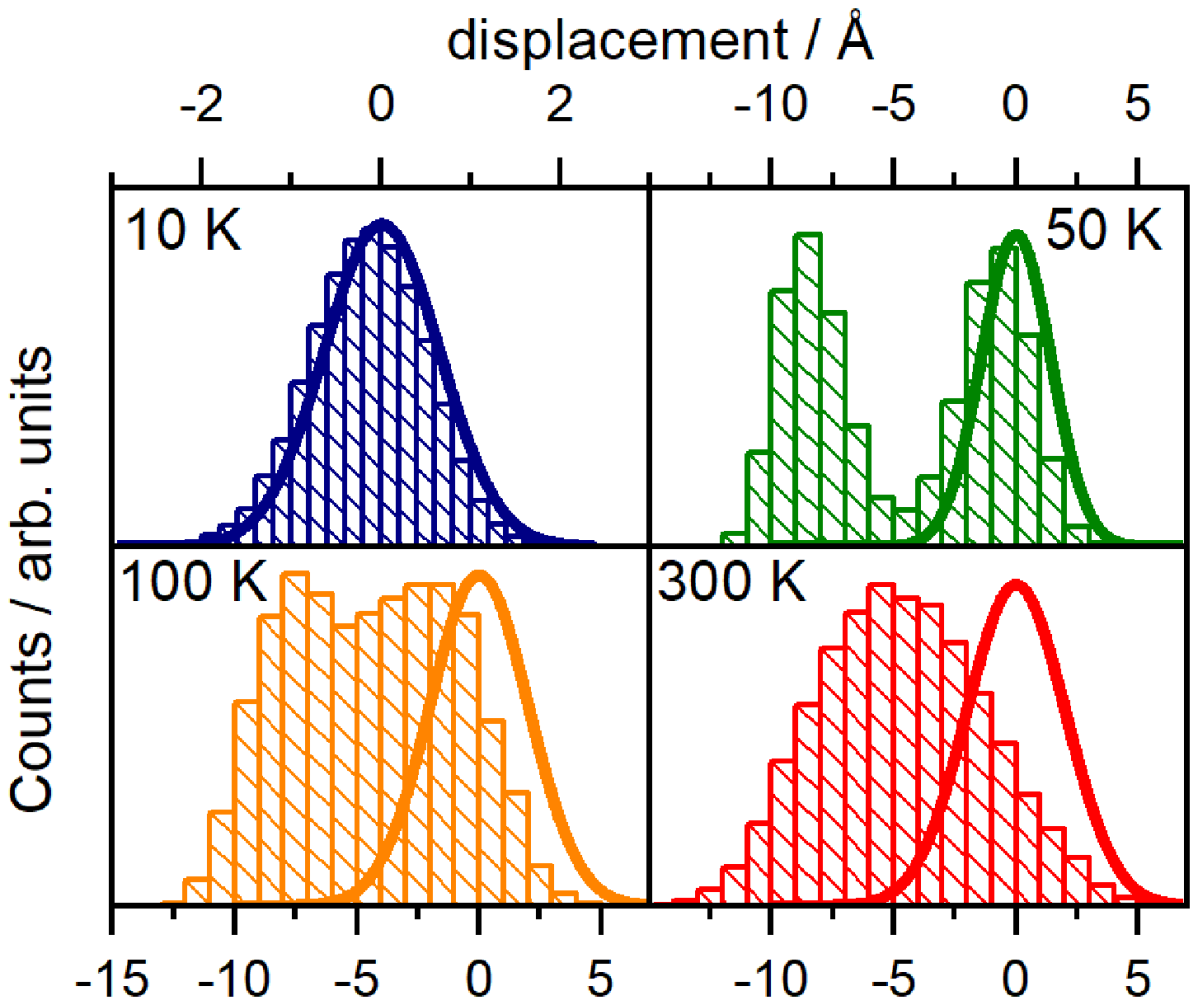
Normal mode displacement probability distribution for selected an harmonic normal mode 3 of the orthogonal dyad. Statistics are derived from 20,000 configurations over 800 ps MD trajectories. Thick lines correspond to a displacement according to a harmonic approximation, adopted in normal mode analysis.

In detail, we recorded the displacements from *R*_0_ geometries at 10, 50, 100, and 300 K and transformed out the translational and rotational motion of the dyad (Amadei et al., 2000). Next, we identified the normal modes of GAFF (MD) potentials, noting that these normal modes correspond well to those obtained from CAM-B3LYP. The statistics of the projection on normal modes were calculated and compared to the Gaussian statistics of approximate (NMA) harmonic dynamics. While statistics of most coordinates including NM1, NM2 (shown in Supplementary Material, section 2) follows a standard harmonic pattern, we found handful coordinates (namely modes 3,15, 39, 45, 64, 67, and 78) with significant deviations. As an example we demonstrate such a behavior by showing the statistics of the third normal mode at various temperatures in Figure 5. At 300 K, the temperature at which the experiments were conducted, the unimodal statistics deviates only moderately from a Gaussian distribution κ_*NM*3_ = 2.67, but are clearly displaced from the global potential minima *R*_0_. Thus the molecular motions are not driven by the harmonic potential explored by NMA, the unimodal character is rather related to the central limit theorem. The distinctly multi-well character appears when MD's are run at lower temperatures: Statistics show two peaks consistent with a double well potential at intermediate temperatures 50–100 K (kurtosis κ = 1.43) −2.05 then becoming centered around the global minima at 10 K, where we finally approach the Gaussian statistics κ = 3.00 of (NMA) harmonic approximation. Nongaussian statistics are demonstrated in Supplementary Material, section 2 also for other modes 15, 39, 45, 64, 67, and 78.

Caution is needed, however, for appropriate interpretation of these non-Gaussian displacement projections, one should not link them straightforwardly to a simple anharmonic potential energy profiles along a single normal mode coordinate (normal modes are approximately harmonic). The double-well type potential type becomes apparent in higher dimensions when more than two normal modes are combined. Accordingly, none of the normal mode show a clear correlation with donor-acceptor coupling *J*. The enhancement of *J* is better linked with combinations of several low-energy normal modes (correlation statistics in Supplementary Material, section 2). In other words, the complex multidimensional potential landscape is the physical source of the observed enhancement of *J*, which is not apparent in a pure NMA.

For the parallel oriented dyad, calculated with the same technical settings, the dipoles of donor and acceptor are parallel in the optimal geometry ***R***_0_. We obtain *J* = −230 cm^−1^, using the TDC method (Equation 6) for the optimal ground state geometry, which is a higher value than predicted in the dipole approximation (*J* = −150cm^−1^; both at ***R***_0_). The distributions of coupling (MD sample of N = 1,000 geometries) in the right panel of Figure 4 is energetically narrow and peaked at *J* = −247cm^−1^, (standard deviation $\sqrt{\langle J^{2}\rangle -{\langle J\rangle }^{2}}=11$cm^−1^). In contrast to the ortho-case, the Pearson kurtosis κ = 3.12 does not significantly exceed the typical Gaussian result considering the sample range and correlation time (see Supplementary Material, section 2). To transcend the sole focus on the fourth moment we have further used the Smirnov-Kolmogorov test (Lilliefors, 1967; Weiss, 1987) but even here no statistically significant difference from the normal distribution was found at the standard confidence level 0.95. The contribution of fluctuations is thus insignificant for the parallel dyad, i.e., the distribution has a rather small standard deviation with respect to the mean, and the coupling obtained by TCD for optimal geometry ***R***_0_ is *J* = −230 cm^−1^. The quantum chemical estimate of the intermolecular coupling in the parallel dyad was not substantially altered by MD sampling of geometries in comparison with the TDC evaluation at optimal geometry (−247 vs. −230cm^−1^). As mentioned in previous section, the value for *J* obtained from fitting the absorption spectrum of the parallel dyad is markedly lower at −200 cm^−1^. We attribute this difference to the fact that DFT-based calculations neglect shielding effects from solvent molecules, and thus tend to overestimate interchromophoric couplings (Hsu et al., 2001; Renger and Müh, 2012).

## 4. Discussion

We have developed a vibronic model of excitonic transport in *peri-*arylene dyads that is applicable to a variety of geometric arrangements and transport regimes. In particular, we studied donor-acceptor dyads in the orthogonal and parallel spatial arrangements.

The Förster regime of transport dynamics was reproduced theoretically within a vibronic model for the orthogonal dyad. This assignment is supported by a flawless reconstruction of the orthogonal dyad's absorption spectrum by its constituents, and furthermore by the reported single-exponential transport within the usual observation window. Previously reported inconsistencies between experimental transport times and microscopic QC parameterizations of a Förster model should thus be untangled from the latter end. The deficiencies of the normal mode approach to thermal fluctuations were overcome by MD sampling of the molecular geometries. The analysis found somewhat complex, but quantitatively adequate fluctuation statistics of intermolecular coupling. Anharmonic potentials of low-frequency modes serve as a physical explanation. One may speculate about alternative models to rationalize the fast transport in the orthogonal dyad. For instance, we tried to estimate the possible mediation through excited states of the linker molecule, or the electron transfer through linker. Due to the unfavorable geometric orientation of the donor, linker and acceptor molecules, we found no electronic state on the linker molecule that would strongly interact with both donor and acceptor. The electron transfer mechanism can also be ruled out because the charge transfer states donor-linker and acceptor-linker are at least 8,000 cm^−1^ higher than the local excited states and their coupling was found in the range of 100 cm^−1^ using a multistate FCD approach (Nottoli et al., 2018) with wB97XD functional.

We also investigated the excited state as a possible source of the orthogonality breaking. However, the results of QC calculations of the excited state exhibit perfect orthogonal arrangement of the transition dipoles for the optimal structure. Also, accounting for an implicit solvent effect using polarizable continuum model of GAUSSIAN (Mohamed et al., 2016) has not altered the optimal geometry nor improves the estimate of *J*.

We thus remain with the complex anharmonic surfaces treated by the explicit MD as the key factor for explaining the high rates of excitation transport in the orthogonal dyad. Closer examination points toward a multi-well picture of the potential landscape for vibrational motions. This is not unprecedented, as similar picture have been advocated to explain temperature-dependent spectral densities, for example in photosynthetic units (Rancova and Abramavicius, 2014). In the same spirit, the geometry statistics of Figure 5 tends toward a unimodal, almost Gaussian distribution at higher temperatures. The distribution's width is, however, different from estimates derived from the surfaces around a global potential minimum.

This observation calls for a cautious inspection of the functional central limit theorem in a physical context: While indeed the composition of many stochastic vibrational coordinates bring the statistics of fluctuations toward the Brownian bridge (Donsker, 1951), and could be thus mapped onto the system of harmonic oscillators at a given temperature (Chernyak et al., 2006), these mappings are themselves temperature dependent. The anharmonic nature of low frequency vibrations is manifested as the need for *ad hoc* corrections to parameterizations of standard harmonic bath models when the temperature dependent optical spectra are studied.

The transport dynamics of the parallel dyad reveal the interplay of excitonic transport and vibrational relaxation. This allows us to track the relaxation pathways within the dyad; the presented vibronic framework is essential for these purposes. For the parallel dyad the vibronic methodology is essential to describe the relaxation pathways within the dyad. The two main factors determining relaxation dynamics are the strong interchromophoric coupling, on the one hand, and resonance between the donor-acceptor gap and the high frequency underdamped stretch mode on the other. Both factors are evident in the absorption spectrum as the levels are shifted and dipoles redistributed among the states in resonance, which means that the linear spectra can not be easily reconstructed from a simple sum of the constituents.

The presented work highlights several possible directions of future research. In general, the study of donor-acceptor systems would benefit from a unified theoretical treatment of exciton transport. Fluorescence labels used to track single protein dynamics by analyzing photon arrival trajectories can be mentioned as an example, where a wide parametric range enters the analysis of excitation quenching (Yang et al., 2003). Largely different couplings also pertains to various stages of light harvesting in photosynthetic complexes.

From a theoretical perspective, a stochastic extension of the presented vibronic model in the spirit of Šanda et al. (2010) and Šanda and Mukamel (2011) promises a profound analysis of dynamical dipoles and their couplings. A more sophisticated inclusion of dynamical dipoles into the vibronic dynamics using a Hamiltonian description applicable at any temperature and its correct MD/DFT microscopic parametrization would be challenging, but would provide a significant test of transport theory.

The couplings from DFT was slightly overestimated compared to the transport data fit due to the neglect of solvent-induced shielding effects in the present DFT. Explicit inclusion of solvent molecules would be a costly, but straightforward way to avoid the extreme of complete neglect or macroscopic scaling for the rather small, compact dyads.

## Data Availability Statement

The raw data supporting the conclusions of this article will be made available by the authors, without undue reservation.

## Author Contributions

TM, JH, and FŠ contributed conception and design of the study. VS performed MD/QC simulations. VP simulated absorption/fluorescence spectra of both dyads and calculated the transport dynamics. HL and AW synthesized dyad. JH measured absorption and fluorescence. FŠ wrote the draft of the manuscript. All authors contributed to manuscript revision, read, and approved the submitted version.

## Conflict of Interest

The authors declare that the research was conducted in the absence of any commercial or financial relationships that could be construed as a potential conflict of interest.
