# Supplementary material for: Anharmonic Molecular Motion Drives Resonance Energy Transfer in peri-Arylene Dyads
Source: Front Chem. 2020 Nov 23;8:579166. doi: 10.3389/fchem.2020.579166 (PMC7732524; doi:10.3389/fchem.2020.579166)
Supplement: Supplementary file 1 [file Presentation_1.pdf]

Supplementary Material

1 MODELLING ABSORPTION LINESHAPES

Absorption measurements are reported in main text for the molecules which precise chemical structure is shown in Fig S1. In the present section we precisely define bath parameters summarized in Table II used to simulate the spectra. We start with spectra of individual *peri*-arylenes. As described in the main text, *peri*-arylene molecules have the stretching mode around $1400\ \mathrm{cm}^{-1}$, which is approximated by anharmonic oscillator (Eq. (10) of the main text)) and treated on equal footing with electronic coordinates forming vibronic states. Below we specify how the modulations of the vibronic eigenenergies by low-frequency vibrations and/or solvent degrees of freedom have been taken into account. These are approximated by a set of harmonic bath coordinates q_α.

$$H_B = \sum_\alpha \left(\frac{\mathsf{p}_\alpha^2}{2\mathsf{m}_\alpha} + \frac{\mathsf{m}_\alpha \omega_\alpha^2}{2} \mathsf{q}_\alpha^2 \right) \tag{S1}$$

The set of oscillatory frequencies ω_α is assumed dense, i.e. can be characterized in terms of smooth densities. Following (Perlík and Šanda, 2017) we consider coupling $c_{Uj\alpha}$ to electronic coordinate responsible for electronic dephasing, and linear $c_{V\alpha}$ and quadratic coupling $c_{W\alpha}$ to the $1400\ \mathrm{cm}^{-1}$ mode responsible for vibrational relaxation. The coupling Hamiltonian thus reads

$$H_{SB} = \sum_{\alpha,j} \omega_\alpha \mathsf{q}_\alpha \left(c_{Uj\alpha} |\Psi_{\mathrm{j},\boldsymbol{R}_0}\rangle\langle\Psi_{\mathrm{j},\boldsymbol{R}_0}| + c_{V\alpha}\tilde{q}_j + c_{W\alpha}\tilde{q}_j^2 \right) \tag{S2}$$

where we shorthanded $\tilde{q}_j \equiv q - d_j|\Psi_{\mathrm{j},\boldsymbol{R}_0}\rangle\langle\Psi_{\mathrm{j},\boldsymbol{R}_0}|$. The effect of these three modulations can be summarized through three spectral densities

$$\begin{aligned}
\xi_U(\Omega) &\equiv \sum_\alpha \frac{\hbar\omega_\alpha}{m_\alpha}(c_{U1\alpha} - c_{U_01\alpha})^2 \delta(\Omega - \omega_\alpha) \\
\xi_V(\Omega) &\equiv \sum_\alpha \frac{\hbar\omega_\alpha}{m_\alpha} c_{V\alpha}^2 \delta(\Omega - \omega_\alpha) \\
\xi_W(\Omega) &\equiv \sum_\alpha \frac{\hbar\omega_\alpha}{m_\alpha} c_{W\alpha}^2 \delta(\Omega - \omega_\alpha)
\end{aligned}$$

which can be arbitrary positive functions. It is common practice to expand them into the sums of Lorentzians $\xi(\Omega) = \sum_j 2\lambda_j\Lambda_j\Omega/(\Lambda_j^2 + \Omega^2)$ each representing a Gaussian-Markovian coordinate with magnitude λ_j and autocorrelation time $1/\Lambda_j$. In the present simulations we consider a single timescale associated with each motion in question, i.e. approximate the spectral densities by a single Lorentzian

$$\xi_U(\Omega) = \frac{2\lambda_U\Lambda_U\Omega}{\Lambda_U^2 + \Omega^2}, \qquad \xi_V(\Omega) = \frac{2\lambda_V\Lambda_V\Omega}{\Lambda_V^2 + \Omega^2}, \qquad \xi_W(\Omega) = \frac{2\lambda_W\Lambda_W\Omega}{\Lambda_W^2 + \Omega^2} \tag{S3}$$

representing an exponentially decaying coordinate (Chernyak et al., 2006; Caldeira and Leggett, 1983; Mukamel, 1995).

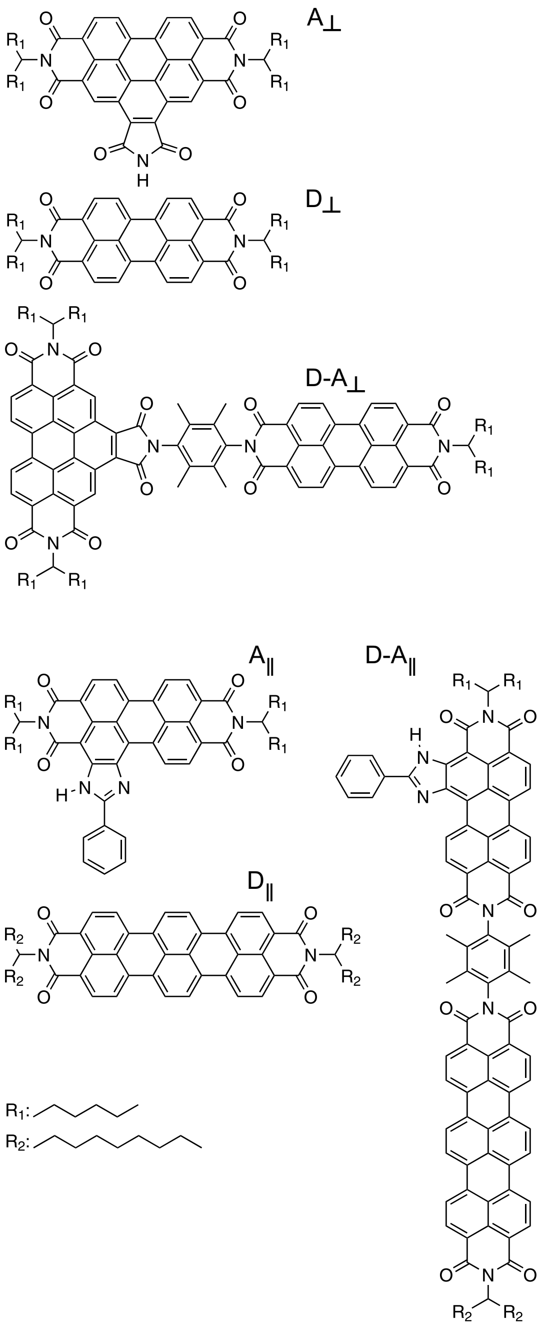

Figure S1. The chemical structures of *peri*-arylenes which spectra are reported in Fig 2 of main text. Long side chains (R_1, R_2) were attached to the molecules to increase solubility (do not enter calculations).

For the dyads, we assume that the condensation of *peri*-arylenes neither significantly changes the constituent electronic excitations, nor the local vibrations. We thus adopt a local type of coupling

$$H_{SB} = H_{SB}^{A} \otimes \mathbb{1}^{D} + \mathbb{1}^{A} \otimes H_{SB}^{D} \tag{S4}$$

where H_{SB}^{A} operates on the acceptor degrees of freedom and similarly H_{SB}^{A} for the donor. In other words, we use the bath parameters obtained from fitting the spectrum of individual *peri*-arylenes (summarized in Table II of the main text) for simulating spectra and the population dynamics of the dyads.

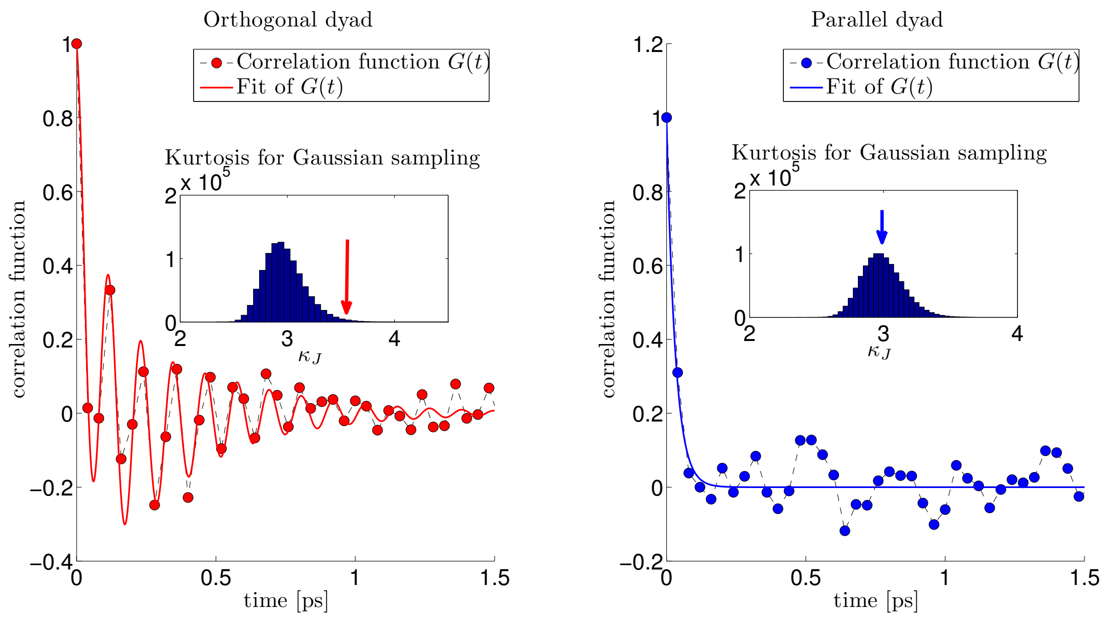

Figure S2. The autocorrelation function Eq. (S5) of coupling statistics for ortho-dyad (left panel) and para-dyad (right panel). Inset: Histograms of kurtosis estimates as sampled (for 120 ps or 40 ps trajectories) from multivariate normal distribution (Chernyak et al., 2006) with correlations given by $G(t)$ obtained from 10^6 MC simulations. Arrows point to the actual value of coupling statistics in Fig 4.

The simulations of dyad spectra and transport dynamics start with diagonalization of the vibronic part of Hamiltonian (see main text), the matrix elements of coupling Hamiltonian (S4) in this vibronic basis are further separated into diagonal and off-diagonal components, which are accounted for by second cumulant and master equations, respectively. The full simulation protocol for the linear and nonlinear response of a vibronic aggregates has been recently published (Perlík and Šanda, 2017), in the present simulations the anharmonicity of the system vibration is allowed along (Galestian Pour et al., 2017).

2 EXTENDED CHARACTERIZATIONS OF THE COUPLING STATISTICS

The MD methodology used to thermalize dyad and thus generate coupling statistics according to Eq. (7) allows also to learn beyond the static properties presented in the main text and visualise also the coupling trajectories $J(t)$. The autocorrelation function ($\langle \ldots \rangle_\tau$ is time average along trajectory)

$$G(t) \equiv \frac{\langle (J(t+\tau) - \bar{J})(J(\tau) - \bar{J}) \rangle_\tau}{\langle (J - \bar{J})^2 \rangle} \tag{S5}$$

for the dyads is shown in Fig S2. The orthogonal case decorrelate within 200 fs and fits well with $G(t[ps]) = 1.13e^{-t/0.113}\sin(\pi(t - 1.062)/0.914) + 0.45e^{-t/0.359}\sin(\pi(t + 0.258)/0.058)$, a damped double harmonic form. The parallel dyad is decorrelated more rapidly within 50 fs without pronounced harmonic modulation and fits well with $G(t[ps]) = e^{-t/0.031}$. We note that the sampled trajectory is by orders longer that the correlation timescales found, as required for the successful simulation.

Found $G(t)$ has been used to adjust statistical tests for the coupling statistics. Inset Fig S2 shows histogram of kurtosis sampled from multivariate normal distribution, which shall be compared to kurtosis actually found for the coupling statistics of dyads at Fig 4.

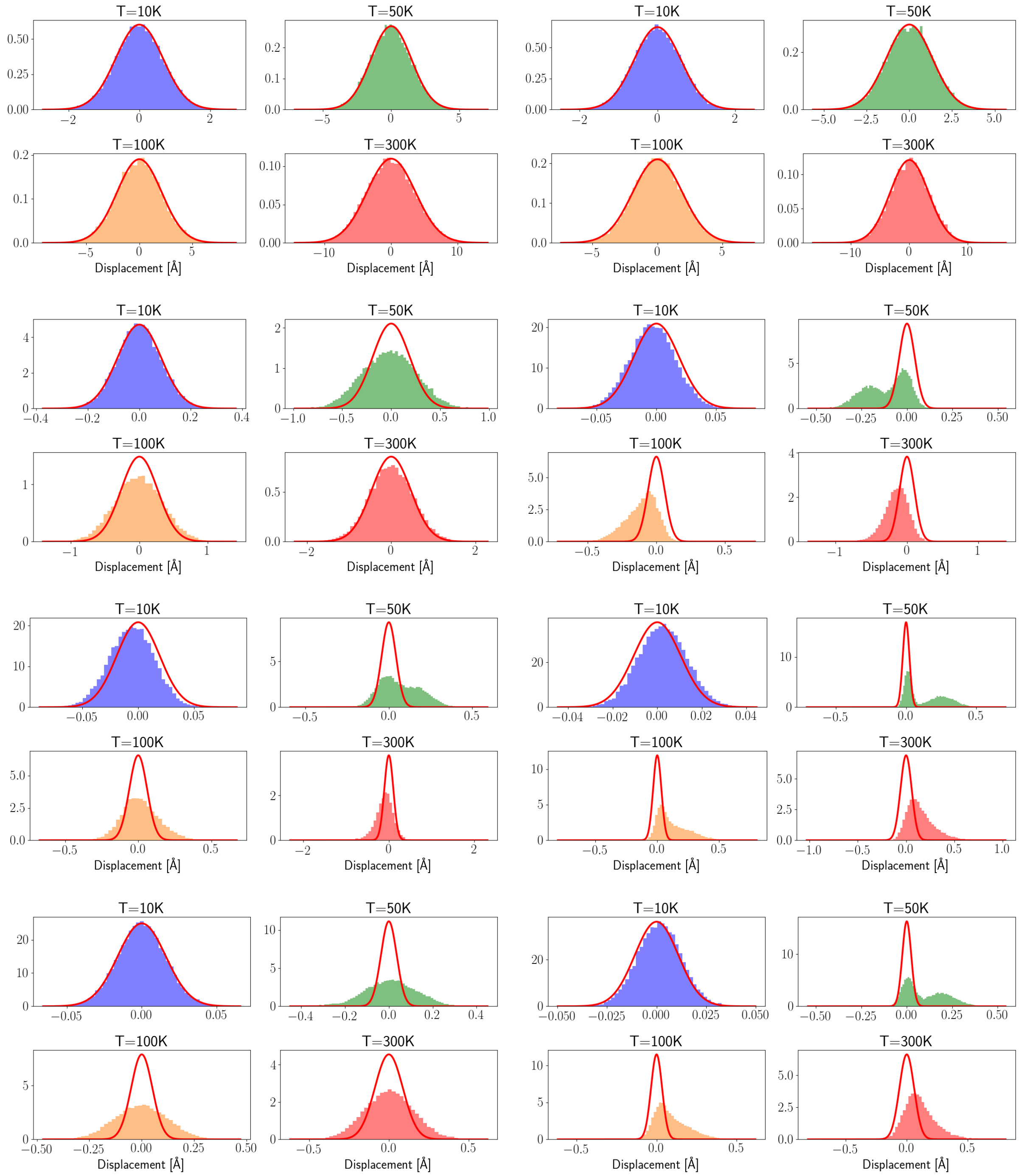

Figure S3. Displacement statistics for normal mode coordinates (left to right and top to bottom) 1,2,15,39,45,64, 67 and 78. The histograms are compared to Gaussian (Boltzmann) distribution expected from a standard (harmonic) normal mode analysis.

We next add supporting evidence for the complex landscape picture of potential advocated in the main text. In Fig S3 we inspected temperature dependent statistics of a few other normal mode coordinates. The lowest two NM1, NM2 and most other modes follow the expected Gaussian profile at all temperatures. Nevertheless still there is a few other modes (in addtion to NM3 treated in the main text), which has atypical profiles beyond any kind of harmonic (Gaussian) approximation for 50 K and higher tempratures.

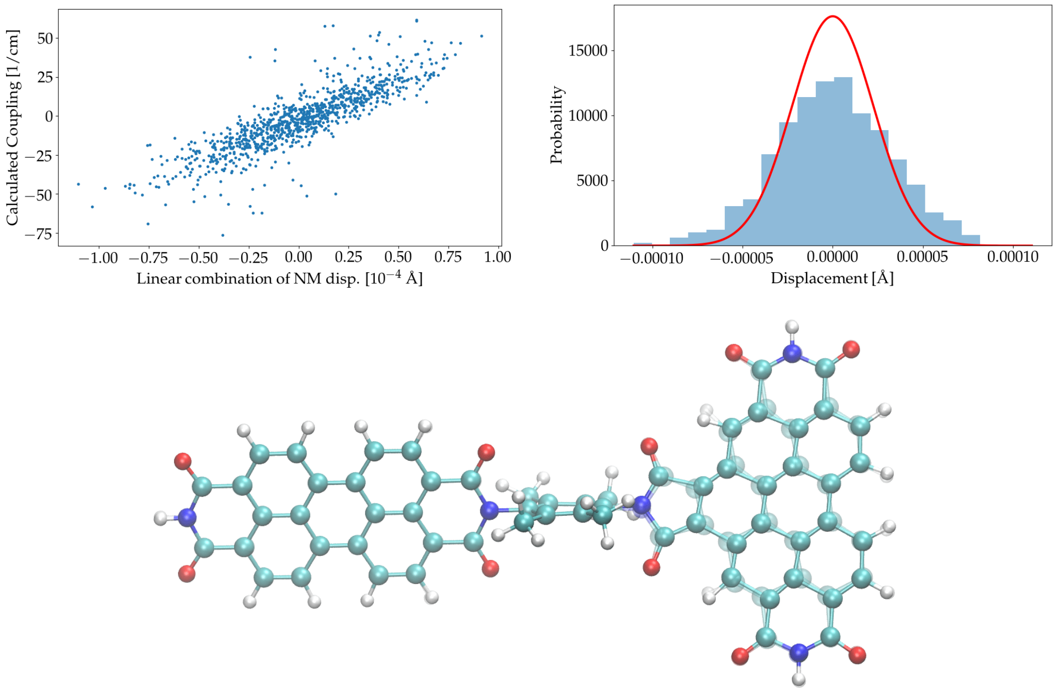

Figure S4. Maximal regression between coordinate and coupling for orthogonal dyad. Regression diagram (left) and displacement statistics at 300 K. Bottom: Visualization of the mode.

Normal modes 39, 64, 78 (in parallel with the NM3 of Fig 5) show bimodal coordinate distribution at 50 K reminding statistics in multi-well potential, which remodels into unimodal, but displaced distributions at higher temperatures. Yet different behavior is observed for normal mode 67. It exhibits an unimodal distribution at all temperatures, but from 50 K it is much wider than the prediction of NMA.

None of these non-Gaussian normal modes is, however, solely responsible for the coupling enhancement effects. In the projections along normal mode coordinates we have not found a simple one-dimensional multi-well potential, and we do not even see any strong correlation between any single normal mode coordinate and coupling (largest covariance has been found for rather high modes NM44 (correlation coefficient $R = 0.27$), NM245 ($R = 0.31$), and NM281($R = 0.49$)). Allowing for arbitrary direction of configuration space, other (not normal) modes were found better correlated with $R = 0.71$ (Fig S4 left). The associated molecular motion depicted in left panel of Fig S4 is composed of many high frequency normal modes twisting the acceptor out of the orthogonal position. The displacement statistics along this mode is profoundly different from Gaussian of NMA (right panel of Fig S4). In other words, the complex multidimensional potential landscape is the physical source of the observed coupling enhancement, which is not apparent in a pure NMA.

3 SYNTHESIS OF PARALLEL DYAD

3.1 Synthesis of 2-(4-amino-2,3,5,6-tetramethylphenyl)-11-(1-nonyldecyl)benzo[13,14]pentapheno[3,4,5-*def*:10,9,8-*d′e′f′*]diisoquinoline-1,3,10,12(2*H*,11*H*)-tetraone

2,3,5,6-Tetramethylbenzene-1,4-diamine (66 mg, 0.40 mmol), 11-(nonyldecyl)-1*H*-benzo[13,14] isochromeno[6',5',4':8,9,10]pentapheno[3,4,5-*def*]isoquinoline-1,3,10,12(11*H*)-tetraone (78 mg, 0.10 mmol) (RN 1041861-64-6P; see (Langhals et al., 2011)), zincacetate (11 mg, 60 μmol) and quinoline (1.8 mL) under argon atmosphere were stirred at 210°C for 4 h (dark blue reaction mixture), allowed to cool, still warm and with vigorous stirring added dropwise to 2 M aqueous HCl (300 mL), stirred for 2 h, allowed to stand overnight, collected by vacuum filtration (D4 glass filter), washed with 2 M aqueous HCl (300 mL), hot distilled water (300 mL) and a mixture of methanol and distilled water (1:1, 300 mL), dried in vacuo (2 d at 110 °C) and purified by column separation (basic alumina, chloroform/methanol 80:1 and then silica gel, chloroform/methanol 40:1). Yield 71 mg (76 %) deep blue solid, m.p.$>$ 300°C. IR (ATR): $\tilde{\nu}$ = 3482 (w), 3398 (w), 2920 (m), 2850 (m), 1693 (s), 1653 (s), 1632 (s), 1584 (s), 1506 (w), 1467 (m), 1437 (m), 1418 (w), 1379 (m), 1351 (s), 1322 (s), 1302 (s), 1250 (m), 1206 (m), 1182 (w), 1139 (w), 1119 (m), 1053 (w), 1009 (w), 854 (w), 841 (m), 806 (s), 790 (m), 750 (m), 721 (m), 694 (w), 680 (w), 668 cm^{-1} (w). ^1H NMR (600 MHz, $CDCl_3$, 27°C, TMS): δ = 8.70 (d, ^3J(H,H) = 8.0 Hz, 2 H, $CH_{arom.terrylene}$). 8.63-8.38 (m, 10 H, $CH_{arom.terrylene}$), 5.25-5.19 (m, 1 H, CH), 3.73 (br, 2 H, NH_2), 2.32-2.24 (m, 2 H, β-CH_2), 2.17 (s, 6 H, 2×CH_3), 2.10 (s, 6 H, 2×CH_3), 1.93-1.86 (m, 2 H, β-CH_2), 1.42-1.13 (m, 28 H, 14×CH_2), 0.83 ppm (t, ^3J(H,H) = 7.1 Hz, 6 H, 2×CH_3). ^{13}C NMR (150 MHz, $CDCl_3$, 27°C): δ = 163.7, 135.9, 135.3, 131.9, 131.0, 130.9, 130.8, 130.3, 129.7, 128.5, 126.2, 125.8, 124.2, 124.1, 121.9, 121.3, 118.9, 54.6, 32.4, 31.9, 29.7, 29.6, 29.3, 27.0, 22.6, 15.1, 14.1, 13.9 ppm. UV/VIS ($CHCl_3$):$\lambda_{max}(\varepsilon)$ = 555.3 (22300), 600.4 (66300), 653.8 nm (130400). Fluorescence ($CHCl_3$): $\lambda_{max}(I_{rel})$ = 673.9 (1.00), 731.3 nm (0.43). Fluorescence quantum yield ($CHCl_3$, λ_{exc} = 600 nm, $E_{600nm,1cm}$ = 0.0322, reference S-13, RN 110590-84-6 with Φ = 0.94): 0.04. MS (DEP/EI) *m/z* (%): 928 (89) [M^+], 661 (66) [M^+ - $C_{19}H_{39}$], 514 (55) [M^+ - $C_{19}H_{39}$ - $C_{10}H_{15}N$]. HRMS ($C_{63}H_{65}N_3O_4$): Calcd. 927.4975, found 927.4984; Δ = 0.0009. $C_{63}H_{65}N_3O_4$ (927.5): Calcd. C 81.52, H 7.06, N 4.53; found C 80.70, H 7.09, N 4.33.

3.2 Synthesis of 2-2-(1-nonyldecyl)-9-ylbenzo[13,14]pentapheno[3,4,5-*def*:10,9,8-*d′e′f′*]diisoquinoline-1,3,10,12(2*H*,11*H*)-tetraone-(2,3,5,6-tetramethylphenyl)-11-(1-hexylheptyl)-5-phenylimidazolo[5',4':5,6]anthra[2,1,9-*def*:6,5,10-*d′e′f′*]diisoquinoline-1,3,10,12(2*H*;11*H*)-tetraone.

2-(4-Amino-2,3,5,6-tetramethylphenyl)-11-(1-nonyldecyl)benzo[13,14]pentapheno[3,4,5-*def*:10,9,8-d'e'f'] diisoquinoline-1,3,10,12(2*H*,11*H*)-tetraone (40 mg, 43 μmol), 9-(1-hexylheptyl)-12-phenyl[2] benzopyrano[6',5',4':10,5,6]anthra[2,1,9-*def*]imidazo[4,5-*h*]isoquinoline-1,3,8,10(9*H*,11*H*)-tetrone (57 mg, 83 μmol) (CAS RN 1402921-10-1; see (Langhals et al., 2017)), zincacetate (7.4 mg, 40 μmol) and quinoline (1 mL) under argon atmosphere were stirred at 230°C for 32 h (color change to dark violet), allowed to cool and still warm added dropwise with vigorous stirring to 2 M aqueous HCl, stirred for 2 h, allowed to stand overnight, collected by vacuum filtration (D4 glass filter), washed with 2 M aqueous HCl (300 mL), hot distilled water (300 mL) and a mixture of methanol and distilled water (1:1, 300 mL), dried in vacuo (1 d at 100°C) and purified by column separation (neutral alumina, chloroform/methanol 100:1, two times silica gel chloroform/methanol 50:1). Yield 4 mg (6 %) bluish black solid, m.p. $>$ 300°C.

R_f ($CHCl_3$/methanol 50:1): 0.8. IR (ATR): $\tilde{\nu}$ = 3412 (w), 2922 (m), 2852 (m), 1696 (s), 1659 (m), 1644 (m), 1625 (m), 1534 (w), 1456 (w), 1414 (w), 1378 (m), 1353 (s), 1329 (s), 1303 (m), 1257 (m), 1205

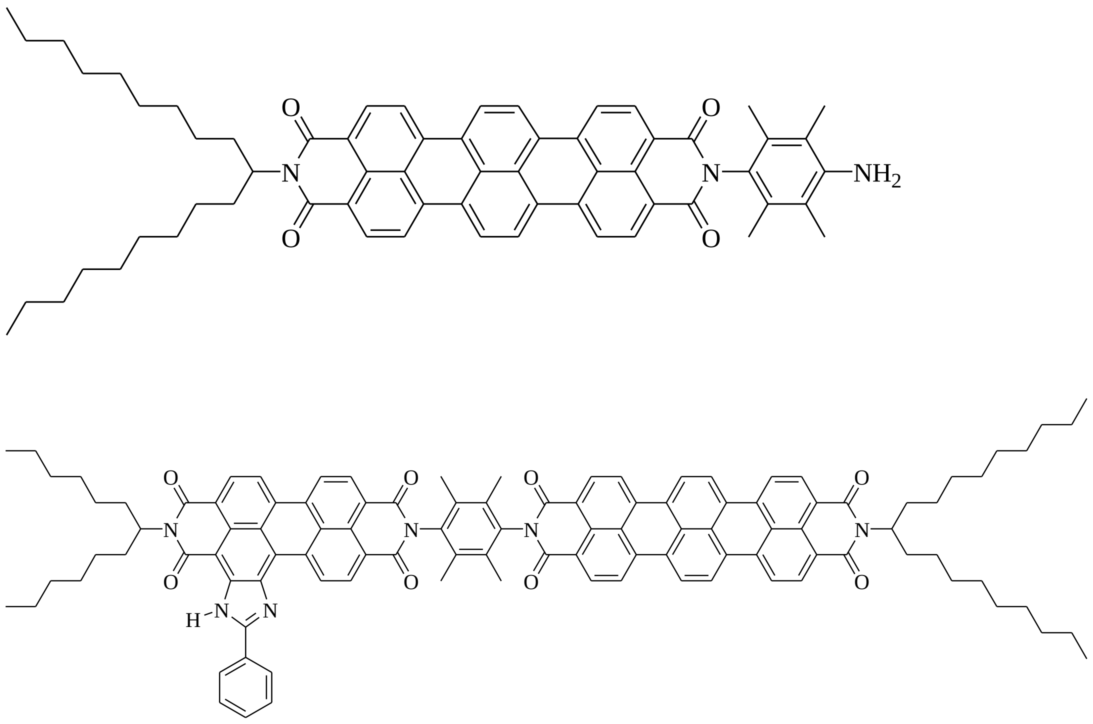

Figure S5. Two steps for synthesising the parallel dyad. Top: Linker substituted toward terylene as described in section 3.1. Bottom: Dyad condensed as described in section 3.2.

(w), 1098 (w), 1016 (w), 842 (m), 809 (m), 748 (w), 686 cm^{-1} (w). ^{1}H NMR (600 MHz, $CDCl_3$, 27°C, TMS): δ = 11.62 (s, 1 H, NH), 10.96 (d, ^3J(H,H) = 8.7 Hz, 1 H, $CH_{arom.}$), 8.98 (d, ^3J(H,H) = 8.0 Hz, 1 H, $CH_{arom.}$), 8.84-8.58 (m, 16 H, $CH_{arom.}$), 8.41-8,40 (m, 2 H, $CH_{arom.}$), 7.69-7.68 (m, 3 H, $CH_{arom.}$), 5.33-5.27 (m, 1 H, CH), 5.26-5.19 (m, 1 H, CH), 2.39-2.26 (m, 4 H, β-CH_2), 2.20-2.17 (m, 12 H, 2$\times CH_3$), 1.98-1.85 (m, 4 H, β-CH_2), 1.60-0.95 (m, 44 H, CH_2), 0.88 (t, ^3J(H,H) = 7.0 Hz, 6 H, 2$\times CH_3$), 0.84 ppm (t, ^3J(H,H) = 7.0 Hz, 6 H, 2$\times CH_3$). ^{13}C NMR (150 MHz, $CDCl_3$, 27.0°C): δ = 170.9, 147.0, 145.6, 138.4, 135.7, 134.2, 132.6, 132.2, 132.0, 129.3, 127.7, 124.4, 121.4, , 37.2, 34.8, 34.4, 34.0, 33.5, 32.4, 31.9, 31.8, 31.7 31.4, 30.2, 29.7, 29.5, 27.3, 26.6, 25.3, 22.7, 22.5, 21.2, 19.7, 15.3, 14.0, 13.7 ppm. UV/VIS ($CHCl_3$): $\lambda_{max}(\varepsilon) =$ 441.0 (10400), 466.5 (11500), 510.2 (14700), 547.7 (43000), 595.9 (101700), 653.8 nm (114800). Fluorescence ($CHCl_3$): $\lambda_{max}(I_{rel}) =$ 671.6 (1.00), 734.0 nm (0.52). Fluorescence quantum yield ($CHCl_3$, $\lambda_{exc} =$ 595 nm, $E_{595nm,1cm}$ = 0.0136, reference S-13, RN 110590-84-6 with Φ = 0.94): 0.92. Fluorescence quantum yield ($CHCl_3$, $\lambda_{exc} =$ 560 nm, $E_{560nm,1cm}$ = 0.0055, reference S-13, RN 110590-84-6 with Φ= 0.94): 0.80. MS (FAB$^+$) *m/z* (%): 1599 [M^+]. MS (MALDI) *m/z*: 1599 [M^+].

REFERENCES

Caldeira, A. O. and Leggett, A. J. (1983). Path integral approach to quantum Brownian motion. *Physica A* 121, 587–616

Chernyak, V., Šanda, F., and Mukamel, S. (2006). Coherence and correlations in multitime quantum measurements of stochastic quantum trajectories. *Phys. Rev. E* 73, 036119

Galestian Pour, A., Lincoln, C. N., Perlík, V., Šanda, F., and Hauer, J. (2017). Anharmonic vibrational effects in linear and two-dimensional electronic spectra. *Phys. Chem. Chem. Phys.* 19, 24752–24760

Langhals, H., Eberspächer, M., and Mayer, P. (2017). Uncatalyzed C-H amination of aromatic compounds under unusually mild conditions with negative enthalpies of activation. *Asian J. Org. Chem.* 6, 1080–1085

Langhals, H., Walter, A., Rosenbaum, E., and Johansson, L. B.-Å. (2011). A versatile standard for bathochromic fluorescence based on intramolecular FRET. *Phys. Chem. Chem. Phys.* 13, 11055–11059

Mukamel, S. (1995). *Principles of Nonlinear Optical Spectroscopy* (Oxford University Press)

Perlík, V. and Šanda, F. (2017). Vibrational relaxation beyond the linear damping limit in two-dimensional optical spectra of molecular aggregates. *J. Chem. Phys.* 147, 084104
